# FPN-Based Faster R-CNN for Fiber Distributed Acoustic Sensing Intrusion Detection in High-Speed Railway

**DOI:** 10.3390/s26092844

**Published:** 2026-05-02

**Authors:** Zhiguang Lei, Zezheng Dong, Hao Xu, Xiao Xiao, Xin’an Qiu

**Affiliations:** 1 Lanzhou Institute of Physics, Lanzhou 730000, China; lzgjxh@163.com (Z.L.); qxa_608@163.com (X.Q.); 2 Shanghai Institute of Satellite Engineering, Shanghai 200240, China; dzz5202026@163.com; 3 School of Telecommunications Engineering, Xidian University, Xi’an 710071, China; 25011211094@stu.xidian.edu.cn

**Keywords:** distributed acoustic sensing, intrusion detection, Faster R-CNN, FPN

## Abstract

With the rapid development of railway and intelligent transportation systems, the construction of security systems along high-speed railways has attracted more and more attention. In this paper, we propose a fiber distributed acoustic sensing (DAS) intrusion detection system to detect and identify the intrusion events that threaten the operational safety of high-speed railways. Firstly, we use the DAS system to collect the optical fiber signals around the high-speed railway. Then we design a window to slide the optical fiber signals along the time axis to form the intensity images with the spatio-temporal signal features. After that, we propose a novel framework that integrates the feature pyramid network (FPN) and the Faster R-CNN to extract the features from the fiber signal intensity images to improve the detection rate and recognition rate of the system for high-speed railway intrusion events. Experimental results indicate that the system can identify five kinds of intrusion events. The average detection accuracy can reach 95.51%, and the F1 score of each intrusion event is above 93% on the real dataset. In addition, the system can identify the background noise interference generated by passing trains, and the detection accuracy is 95%, which can significantly reduce the false alarm rate.

## 1. Introduction

The rapid development of railway and intelligent transportation system can significantly improve people’s lives. In recent years, high-speed railways featuring an operating speed of more than 300 km per hour have developed rapidly, creating great convenience for people to travel. According to statistics, as of the end of 2021, China’s high-speed railway operation has more than 40,000 km, ranking among the top in the world. With the rapid development of the railway [1], the safe operation of the high-speed railway has been placed in a critical position, and the monitoring of the high-speed railway perimeter is becoming one of the urgent issues to ensure security [2]. Facing the challenges of long-distance, long-period, and complex environmental conditions, the high-speed railway perimeter detection system must remain sensitive to events, maintain low false alarm rates, and provide accurate alarms; specifically, it should demonstrate excellent environmental robustness by maintaining an average detection accuracy of no less than 90% under various complex noise conditions, such as intense train vibrations and heavy rain. However, the existing technologies have diverse problems in railway perimeter detection. Specifically, the camera-based high-speed railway perimeter detection system is susceptible to the influence of light and visibility. In 2025, the average unit price of global railway track sensors was approximately $5200 per unit. The effective detection range of microwave radar typically ranges from several tens to hundreds of meters. Given the monitoring requirements of high-speed railways that often extend to dozens of kilometers, radar needs to be densely deployed along the lines. Consequently, the deployment cost of microwave radar is relatively high, and the signal fluctuates wildly during long-term operation, resulting in inaccurate detection results. As for the infrared detection technology, it has a high rate of false alarms in bad weather conditions.

To evaluate the performance of various railway monitoring technologies, a comparative summary is presented in Table 1. Compared with traditional sensors, the proposed DAS system provides a superior balance between coverage and cost-effectiveness.

Unlike the above methods, the fiber distributed acoustic sensing (DAS) system has a simple structure, high positioning accuracy, and high sensitivity to weak external vibration signals. In addition, the fiber optic material has the advantages of anti-magnetic interference, anti-radiation, corrosion resistance, and light weight, which is especially suitable for long-period perimeter security monitoring tasks [6]. As a reliable DAS technology, phase-sensitive optical time domain reflectometry (ϕ-OTDR) has the potential of fully distributed vibration monitoring along the optical fiber cable. It can detect the dynamic strain change of the optical fiber caused by external vibration [7,8,9,10,11]. However, most of the existing high-speed railway optical fiber intrusion detection systems can only monitor the signal strength and cannot identify the type of abnormal intrusion events. Besides, some intrusion event identification models usually choose the signal strength, variance and short-time zero crossing rate in the time domain as the features of intrusion events [12,13,14]. These features cannot fully represent the complexity of actual intrusion events and may affect the accuracy of the event identification. Therefore, the high-speed railway intrusion detection is still a challenging problem in the high-speed railway environment.

In response to the above challenges, many scholars have proposed solutions from different perspectives. Zhong et al. [15] constructed an eight-pulse-width-multiplexed ϕ-OTDR system to reduce the missing alarm rate. Wu et al. [16] proposed a novel intrusion signal processing method based on ϕ-OTDR longitudinal time sequence to solve the high false positive rate of ϕ-OTDR. Sun et al. [17] introduced a new feature extraction method for intrusion event identification, which can improve the identification accuracy of the monitoring system and reduce the computation time. Wu et al. [18] further studied the ϕ-OTDR technology and proposed an effective time-domain signal separation and determination method to improve the detection performance of the ϕ-OTDR in complicated environments. In the best case, the signal-to-noise ratio can be improved to about 35 dB. However, the improvements in the above studies do not fully utilize the information of the fiber optic sensors, as only the Rayleigh backscattering intensity information from the fiber optic sensors is utilized. Consequently, they are insufficient for the scenarios requiring fast response capability and high precision. In such scenarios, “fast response” necessitates an end-to-end processing latency of less than 40 ms per frame to ensure real-time alerting, while “high precision” demands a detection accuracy (Precision) exceeding 93% to minimize costly false alarms. Our proposed method achieves an inference time of 36.4 ms (27.5 FPS), which is 75% faster than the standard Faster R-CNN [19]. Simultaneously, it maintains a superior *F*_1_-score of up to 97.44% for critical events like “Excavate”, fulfilling the rigorous safety standards of railway perimeter monitoring.

With the continuous development of deep learning, some researchers have recently applied deep learning technology in the DAS system to improve the identification and classification accuracy of abnormal intrusion events. Wu et al. [20] used a 1D convolutional neural network (CNN) and support vector machines (SVM) to identify five kinds of vibration signals in the tubing monitoring system. However, since DAS signals possess both temporal continuity and spatial distribution in terms of their physical properties, the key features required for classification can only be accurately captured on a spatiotemporal two-dimensional scale. Therefore, labeling each frame of data as an independent vibration signal category is challenging. Therefore, the system may be subject to the interference from other types of vibration signals. Aktas et al. [21] proposed a novel signal processing technology for the fiber DAS system, using the deep CNN to identify threat behaviors. It can achieve more than 93% classification accuracy and the range up to 40 km. Li et al. [22] established the convolutional long short-term memory (ConvLSTM) neural network, using the network to classify the collected distributed fiber optic sensing signals. The intrusion threat detection rate is 85.6%. However, this method cannot effectively distinguish the intrusion signals from noise and other background signal interferences, which can easily cause false positives. Shiloh et al. [23] modeled an optical DAS system with high accuracy and used the generative adversarial network (GAN) to convert simulation data into accurate real data. Then a classification model was established using the VGG16 network. Furthermore, the integration of advanced real-time detectors has significantly pushed the boundaries of railway security. Recently, a notable advancement is the Improved RT-DETR framework, which utilizes a transformer-based encoder-decoder architecture to achieve high-performance railway obstacle detection while maintaining superior real-time efficiency [24]. By optimizing the cross-scale feature interaction, this improved RT-DETR addresses the inherent redundancy and multi-scale detection challenges in high-speed rail environments. Besides, the MSIA-YOLOv8 model has been recently proposed to address the challenges of detecting multi-scale obstacles under adverse weather and low-light conditions, further integrating risk assessment through the DALNet architecture [25]. While these visual-based SOTA models demonstrate exceptional precision in identifying physical obstacles on tracks, their performance remains susceptible to environmental occlusions and limited field-of-view in long-distance longitudinal monitoring. In recent years, the integration of advanced deep learning architectures has revolutionized railway infrastructure health monitoring. A significant milestone is the development of Rail-STrans, an improved Swin Transformer-based method designed for rail surface defect segmentation [26]. By leveraging a hierarchical Vision Transformer with a shifted window mechanism, Rail-STrans effectively captures long-range dependencies and intricate spatial details of rail surface damage, achieving superior performance in railway-specific semantic segmentation tasks.However, while Transformer-based models like Rail-STrans excel in processing static high-resolution visual images for surface inspection, the detection of perimeter intrusions via Fiber Distributed Acoustic Sensing poses a different set of challenges. DAS signals, once converted into spatio-temporal intensity images, are characterized by dynamic vibration patterns rather than static structural textures. Furthermore, these patterns are often corrupted by the intense, non-stationary background noise of operating high-speed trains. This necessitates a robust feature fusion strategy that can handle multi-scale temporal dynamics.

In this paper, we present a high-precision fiber-optic DAS intrusion detection system and validate the detection performance of the system using the real dataset. Distinct from the direct application of computer vision algorithms, our framework introduces a domain-specific structural adaptation to bridge the gap between 1D fiber-optic physical signals and 2D deep learning architectures. By re-purposing the hierarchical layers of the Feature Pyramid Network (FPN), we specifically align the multi-scale feature fusion mechanism with the physical propagation characteristics and frequency distribution of railway intrusion events, thereby enhancing the model’s sensitivity to subtle vibration signatures amidst intense non-stationary noise. Firstly, the DAS system is used to collect the fiber optic signals. Then the collected fiber optic signals are processed in the temporal and spatial dimensions to form the intensity images with the spatio-temporal signal features and input to the detection algorithm model. Finally, the improved Faster R-CNN algorithm based on the feature pyramid network (FPN) is used to detect and identify five kinds of high-speed railway perimeter intrusion events (including train background noise interference, thorn cage pulling, climbing, excavating, and wall chiseling). It improves the detection accuracy of the system and achieves the state-of-the-art (SOTA) result.

## 2. High-Speed Railway Intrusion Detection System Based on DAS

**Figure 1:**
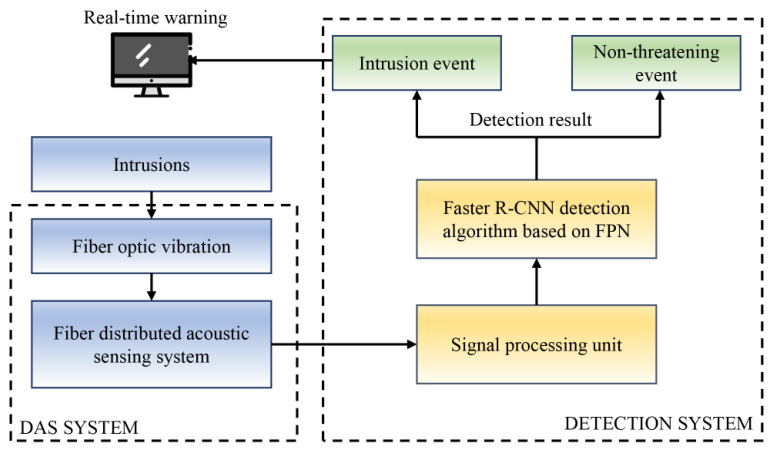
The composition of the intrusion detection system for high-speed railway.

The high-speed railway intrusion detection system, as shown in Figure 1, is mainly composed of the DAS system and the detection system. First of all, we use fiber optic DAS system to monitor and collect fiber optic acoustic signals, and then send the collected fiber optic acoustic signals to the signal processing unit. After that, we propose the improved Faster R-CNN detection algorithm based on the FPN to efficiently detect and classify the processed signals and provide real-time warning after identifying abnormal intrusion events. Section 2 mainly introduces the DAS system. The detection system of the improved Faster R-CNN based on the FPN is described in Section 3.

### 2.1. Phase-Sensitive Optical Time Domain Reflectometry System

In this paper, the DAS system is based on the phase-sensitive optical time-domain reflectometry (ϕ-OTDR) [27,28,29]. The standard ϕ-OTDR needs to inject the pulse light with strong coherence and high-frequency stability into the end face of the fiber as the detection signal. This light source will send out a highly stable laser, and the continuous laser will form a certain width of light pulse after external modulation. The Rayleigh backscatter (RB) signal within one pulse width will interfere during the light pulse propagation along with the fiber. The scattered light interference results can be obtained by detecting the RB light in the pulse range with a suitable photodetector.

**Figure 2:**
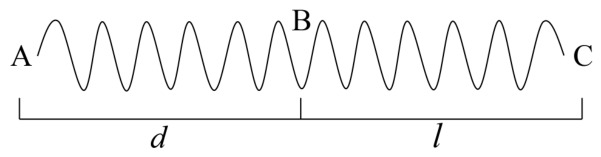
Schematic diagram of interference in ϕ-OTDR distributed optical fiber.

As shown in Figure 2, it is a continuous sine wave generated by the laser after modulation, *d* is the distance between AB and *l* is the distance between BC. Assuming that the time interval of sine wave signal propagation between AC is *T*, The sensing principle of ϕ-OTDR can be described as follows [30]:

(1) $$T=\frac{(d+l)n}{c}$$

where *n* is the refractive index of the fiber core and *c* is the velocity of light in vacuum. The vibration equations at A and C can be expressed as [30]:

(2) $$\left\{\begin{array}{c} y_{A}=\cos\left(w_{A}t\right),w_{A}=2\pi f_{A}\\ y_{C}=\cos\left(w_{C}t\right),w_{C}=2\pi f_{C} \end{array}\right.$$

where $w_{A}$ and $w_{C}$ represent the angular frequencies of point A and point C, $f_{A}$ and $f_{C}$ represent the frequencies of point A and point C, and *t* represents the signal propagation time.

The coherent interference occurs when the scattered light from point A to point B and the scattered light from point C are reflected back to the photodetector at the same time. Therefore, the interference light intensity at point B is the superposition of the pulse light which propagates equidistant before and after point B or the interference light which is reflected to point B. Assuming d=l, the Rayleigh backscattered light vibration equations at A and C then can be given by [30]:

(3) $$\left\{\begin{array}{c} y_{A}^{\prime }=K\cos\left(w_{A}t+\varphi \right)\\ y_{C}^{\prime }=K\cos\left(w_{C}t+\varphi \right) \end{array}\right.$$

where φ is the additional phase of backscattering and *K* is the intensity coefficient of backscattering light. According to the above analysis, the interference equation generated when A and C return to the detector can be expressed as:

(4) $$\begin{array}{cc} y & =y_{A}^{\prime }\left(t\right)+y_{C}^{\prime }\left(t\right)\\ \; & =2K\cos\left(\frac{w_{A}t+w_{C}t}{2}+\varphi \right)\cos\left(\frac{w_{A}t-w_{C}t}{2}\right) \end{array}$$

Ideally, we can assume that $w_{A}=w_{C}$, then Equation (4) can be rewritten as:

(5) $$\begin{array}{cc} y & =2K\cos(w_{A}t+\varphi )\\ \; & =2K\cos\left(\frac{w_{A}t+w_{C}t}{2}+\varphi \right) \end{array}$$

Based on Equation (5), we can obtain the detected optical power, shown as:

(6) $$W=\int y^{2}dt=2K^{2}$$

When there is an intrusion at a certain position of the optical fiber, the refractive index of the fiber will change accordingly, thus causing the phase change of the Rayleigh scattering interference signal in the fiber core. The Rayleigh scattering signal within one pulse width will interfere, which is transformed into the change of the interference signal intensity obtained by the photodetector. The ϕ-OTDR can obtain the change quantity by subtracting the two adjacent detection results of the photodetector, so as to realize the detection of intrusion signals.

### 2.2. Distributed Acoustic Sensing System

**Figure 3:**
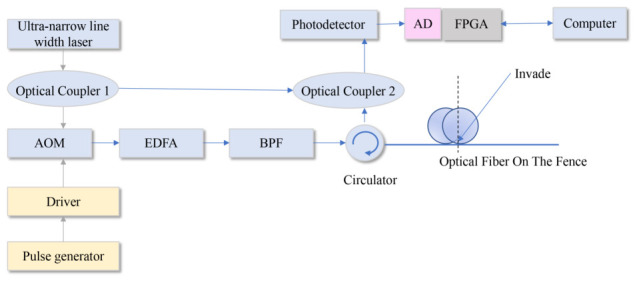
DAS system based on ϕ-OTDR.

The structure of the DAS system based on ϕ-OTDR used in this paper is shown in Figure 3. The key laboratory device models and their corresponding parameters are summarized as follows. A narrow-linewidth ultra-low noise laser was employed, operating at a central wavelength of 1550.12 nm with a linewidth of less than 3 kHz to ensure high phase stability. An acousto-optic modulator (AOM) modulated the continuous light into pulses with a width of 100 ns, providing a spatial resolution of 10 m. A 10 km standard single-mode fiber (SMF-28) was used as the sensing medium, deployed along the high-speed railway test section. The Rayleigh backscattered light was captured by a high-gain balanced photodetector (BPD) with a bandwidth of 200 MHz. The Ultra-narrow line width fiber laser is first divided into two portions by the Optical Coupler (OC) 1, which is modulated to pulse after passing through the Acoustic-Optic Modulator (AOM). Due to the low duty cycle and low power of pulse light, it needs to be amplified by Erbium-Doped Fiber Amplifier (EDFA) and transferred to the Band Pass Filter (BPF) to suppress the amplified spontaneous emission noise. Pulsed light is injected into the sensing optical fiber through the Circulator and transmitted along with the fiber. Part of the light split by OC 1 is used in the local oscillator, which is mixed with the RB light signal via OC 2. Whereafter, the mixed light is detected by the Photodetector, which contains the phase information of the RB light signal. Finally, the Analog to Digital (AD) chip samples the RB signal, whose sequence is controlled by the Field Programmable Gate Array (FPGA). The amplitude-frequency and phase-frequency characteristics of mixed signals can be obtained through the Fast Fourier Transform (FFT). FPGA processes and calculates the detection signals to get the phase difference of the signal when the fiber optic sensing is not intruded and has been intruded, and transmits it to the Computer side to obtain the position of the intrusion signal.

**Figure 4:**
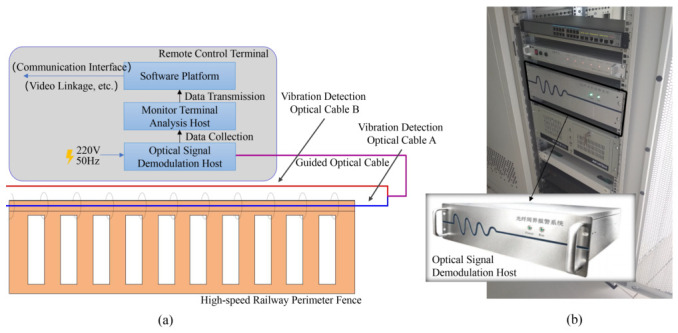
(**a**) High-speed railway intrusion detection system based on DAS; (**b**) Laboratory equipment.

Figure 4a shows the overall structure of the intrusion detection system based on DAS, and Figure 4b shows the laboratory equipment. The intrusion detection system consists of the following four parts.

- **Vibration Detection Optical Cable:** The vibration detection optical cable is used to transmit fiber optic signals around the high-speed railway perimeter. A standard single-mode tactical optical fiber (SMF-28e) is utilized as the distributed sensing medium. The cable is protected by a reinforced aramid yarn layer and a flame-retardant LSZH (Low Smoke Zero Halogen) jacket, specifically designed to ensure high sensitivity and durability along the high-speed railway perimeter.
- **Optical Signal Demodulation Host:** The optical signal demodulation host is responsible for the modulation, amplification and reception of the optical signals. This unit integrates a narrow-linewidth fiber laser (wavelength: 1550.12 nm, linewidth: <3 kHz), a high-speed acousto-optic modulator (AOM), and an Erbium-doped fiber amplifier (EDFA). It is responsible for modulating continuous light into 100 ns pulses and pre-amplifying the weak Rayleigh backscattered signals.
- **Monitoring Terminal Analysis Host:** The monitoring terminal analysis host is mainly used for vibration signal acquisition, processing and communication with the software platform. This component is an industrial-grade workstation equipped with a high-speed data acquisition card (DAQ, sampling rate: 200 MS/s, 14-bit resolution). It performs real-time IQ demodulation and provides the raw data for subsequent signal processing and phase-space reconstruction.
- **Software Platform:** The software platform is responsible for storing and analyzing signal data, implementing the detection algorithm and providing early warnings. The platform is developed using a C++17/Python 3.9 hybrid architecture. It leverages the PyTorch 2.0 framework for deploying deep learning models for real-time event classification. The system features a custom GUI dashboard for visualizing spatial-temporal heatmaps and managing millisecond-level early warning alerts.

## 3. FPN-Based Faster R-CNN for Intrusion Detection

### 3.1. Sliding Window Spatio-Temporal Image

**Figure 5:**
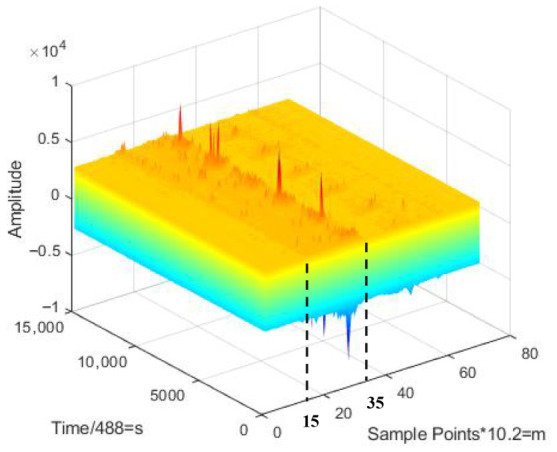
An example of fiber optic acoustic signal data.

Figure 5 shows a climbing intrusion event’s fiber-optic signal image, containing the signal amplitude, sampling distance point, and sampling time point information. The horizontal axis represents the spatial index, while the vertical axis denotes the slow-time index. The color-coded legend indicates the normalized amplitude of the vibration signal, where warmer colors represent higher energy concentrations—indicative of an intrusion event—and cooler colors represent the ambient background noise. The *X*-axis represents the number of sampling distance points, and every 10.2 m of fiber represents one sampling point. The selection of a 10.2 m spatial sampling interval is intended to strike a balance between spatial granularity and the computational efficiency of the FPN-based model. The *Y*-axis represents the number of sampling time points, and 488 time points represent one second. In Figure 5, the Z-axis represents the amplitude of the monitored signal, which reflects the intensity of external vibration experienced by the optical fiber at a specific position and time. When an intrusion occurs at a certain location, the refractive index at that position changes, causing significant fluctuations in the interference signal intensity. The abnormal amplitude observed between sampling points 15 and 35 in Figure 5 serves as the key basis for identifying the “climbing” intrusion event.

**Figure 6:**
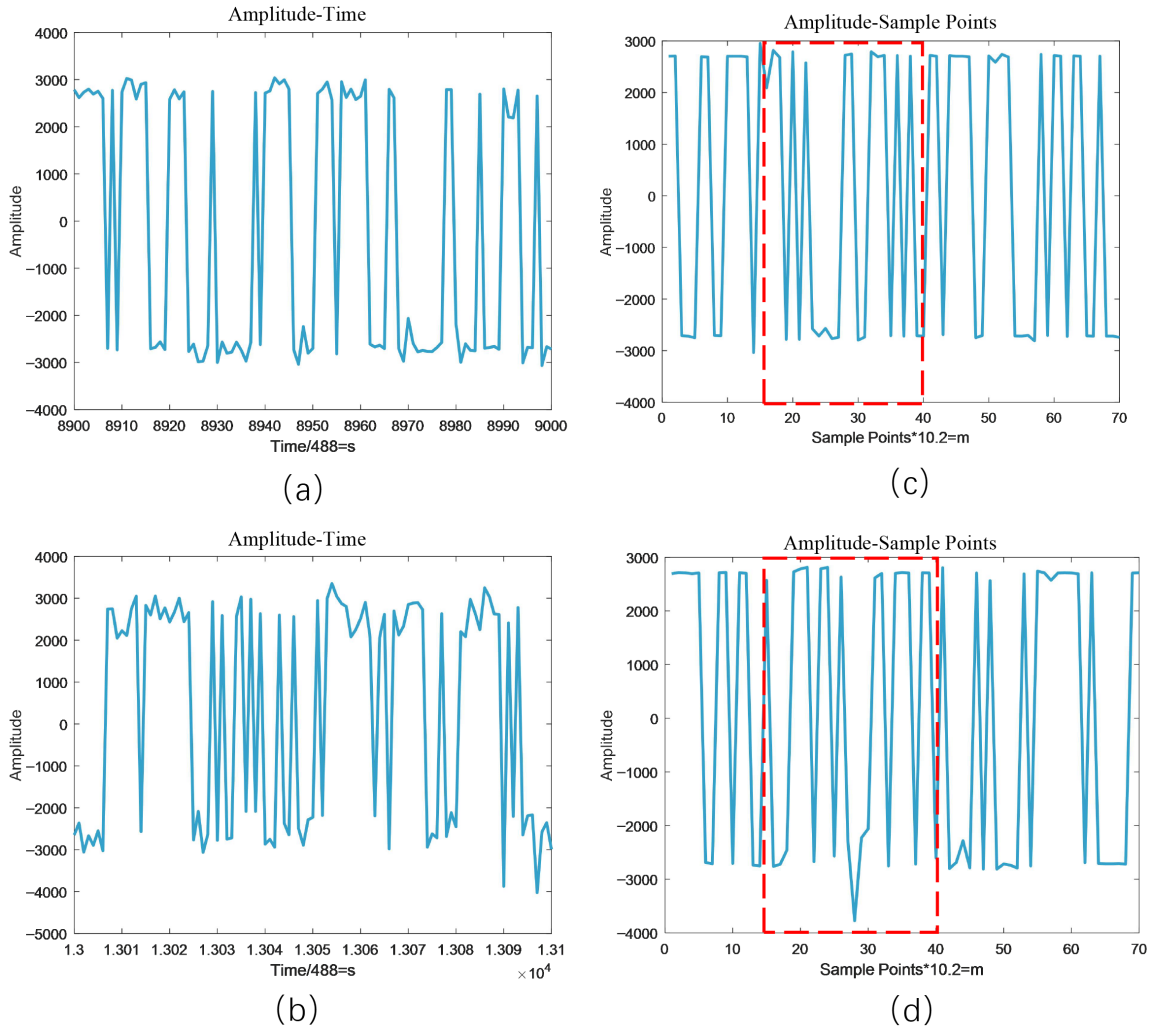
An example of the acquired data ((**a**,**c**): The no-intrusion event. (**b**,**d**): The climb-intrusion event).

For each sampling point, the optical fiber acoustic signal data collected at different time points are dynamically relevant. Therefore, how to effectively extract the spatio-temporal correlation of the signals is an important step for signal identification. Figure 6a,b represent the time-domain waveforms of the signal detected at one sampling point during a period of time when no-intrusion event and climbing intrusion event occurred, respectively. The horizontal coordinate represents the sampling time points and the vertical coordinate indicates the amplitude of the vibration acoustic signal monitored by the system at the corresponding time. Due to subtle changes in the local optical fiber refractive index caused by intrusions, the phase of the backscattered light at that point becomes modulated. Under the coherent superposition effect, this phase modulation is transformed into intensity fluctuations of the Rayleigh Backscattering profile along the time axis. As shown in Figure 6, at sampling point X, the envelope of the RB trace no longer maintains temporal stability but exhibits random modulation characteristics correlated with the intrusion frequency. Figure 6c,d represent the signal waveforms detected at different sampling points when the no-intrusion event and the climbing intrusion event occurred, respectively. The red rectangular boxes have illustrated how intrusion and non-intrusion events cause variations in signal waveforms across various sampling distances. Unlike Figure 6a,b, the horizontal coordinates represents the number of sampling distance points. From Figure 6, we can see that the non-intrusion event and the climbing intrusion event have different signal waveform features. However, the difference between intrusion events and the non-intrusion event is not obvious, and it is difficult to distinguish them in general detection algorithms.

**Figure 7:**
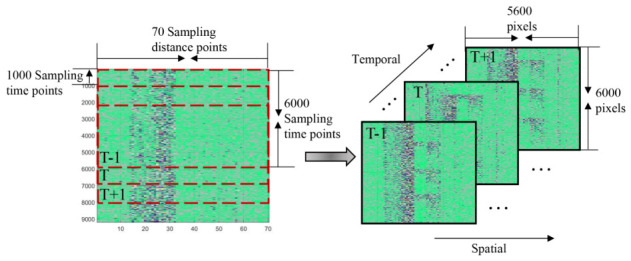
The sliding window of optical fiber signal data.

**Figure 8:**
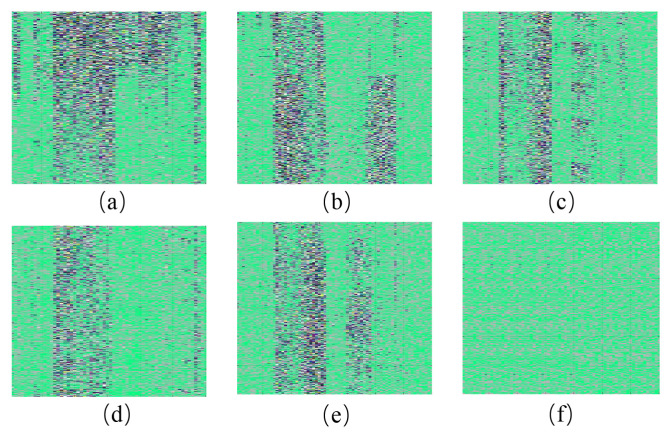
Spatio-temporal signal feature intensity images of the six kinds of fiber signals, from (**a**–**f**): train-interference, pulling-thorn-cage, climb, chiseling-wall, excavate, no-intrusion.

To address the above problems and comprehensively analyze the spatio-temporal features of the intrusion signals, we project the map in Figure 5 onto a plane to form the spatio-temporal feature intensity image. Unlike ref. [22], we design a window to slide the optical fiber acoustic signals along the time axis. As shown in Figure 7, the window contains fiber-optic acoustic signal data with 6000 sampling time points and 70 sampling distance points. We slide the fiber optic acoustic signals in steps of 1000 sampling time points. Then, the optical fiber signal in each sliding window forms an image. The sliding window size is set to 6000 sampling points, which corresponds to a duration of approximately 12.3 s based on the system’s sampling frequency of 488 Hz. The selection of this value is primarily dictated by the physical characteristics of typical intrusion events, such as climbing, digging, and wall-breaking. Since these activities generally exhibit periodic or continuous patterns, a 12.3-s window is sufficient to capture one or more complete cycles of vibration evolution, ensuring that the spatio-temporal feature maps retain adequate semantic information for deep-learning-based identification. Furthermore, a sliding step of 1000 sampling points is adopted, resulting in an 83.3% overlap between adjacent windows. This configuration serves to implement data augmentation and ensures that intrusion signals occurring at arbitrary moments are fully encapsulated within the window, thereby preventing missed detections caused by signal truncation at window boundaries. Simultaneously, it maintains an efficient real-time response frequency for the alarm system. To make the proportion of images more fitting, we perform linear interpolation on the horizontal direction of the images. The final images are 5600 pixels in width and 6000 pixels in high. Among them, the vertical coordinate is the sampling time points of each group of fiber optic acoustic signals. The horizontal coordinate is the sampling distance points. Different colors indicate the amplitude magnitude of the fiber optic acoustic signal. Compared to the method in [19], linear interpolation makes the generated spatio-temporal intensity maps more suitable for processing by deep learning networks. It also enhances the detail information within the images, thereby providing richer and higher-resolution input for the subsequent FPN structure. This signal processing method expands the number of samples of labeled intrusion signals in the dataset and increases the color information of the images and the differentiation of the intrusion signals. As shown in Figure 8, the spatio-temporal signal feature intensity images of different intrusion events are significantly different.

### 3.2. FPN-Based Faster R-CNN Detection Algorithm

**Figure 9:**
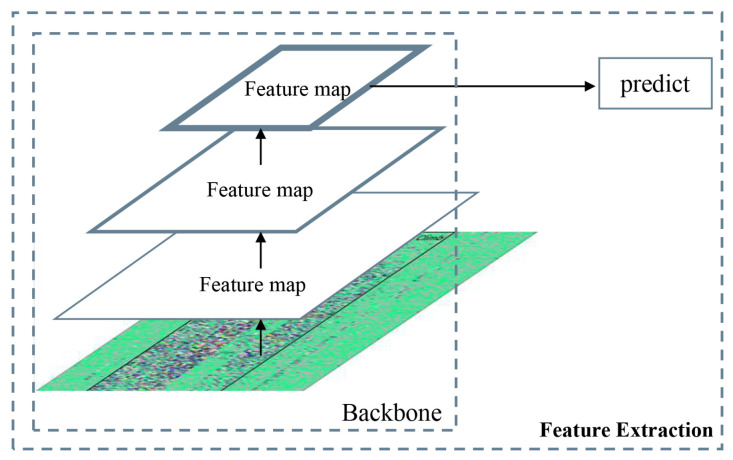
Single feature map prediction (Faster R-CNN).

Faster R-CNN [31] is an object detection algorithm which combines region recommendation and CNN. It has excellent detection effect in complex scenes [32,33,34,35,36,37]. The algorithm uses feature extraction network, region proposal network (RPN) and classification and regression network to achieve end-to-end object detection. However, when the Faster R-CNN algorithm deals with multi-scale objects detection, the CNN only acts on the feature map generated at the last layer. As shown in Figure 9, the Faster R-CNN only uses a single deep feature map to predict objects. This method will make the model ignore much detailed information of the input image, resulting in inadequate feature extraction and affecting the detection accuracy of the system.

**Figure 10:**
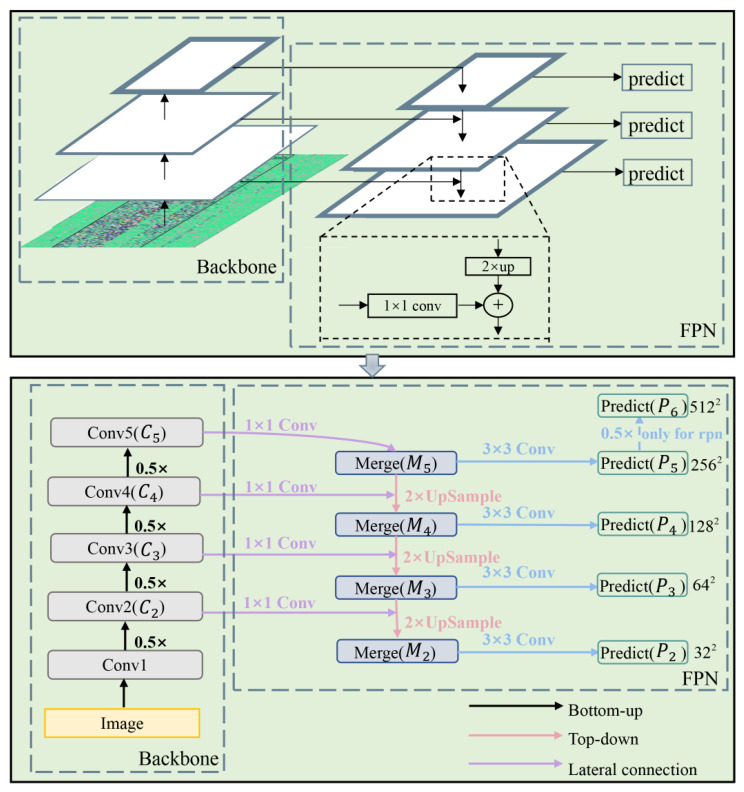
Feature pyramid network.

To improve the above shortcomings, we introduce the feature pyramid network (FPN) [38] into the Faster R-CNN algorithm. FPN is a top-down network structure with lateral connections, which is used to construct feature maps of different sizes with high-level semantic information. Figure 10 shows the structure of the FPN, and the bottom diagram is a further explanation of the top diagram. As shown in the lower diagram of Figure 10, the feature map of layer $C_{5}$ is first performed by 1 × 1 convolution, and the number of channels of the feature map is changed to obtain the feature map of layer $M_{5}$. The feature map of layer $M_{5}$ is up-sampled to obtain a feature map of the same size as the feature map of layer $C_{4}$. This feature map is added to the feature map of layer $C_{4}$ after 1 × 1 convolution to obtain the feature map of layer $M_{4}$. The same process is done twice to get the feature maps of layer $M_{3}$ and layer $M_{2}$ respectively. After that, the $M_{2}$, $M_{3}$, $M_{4}$, and $M_{5}$ layer feature maps are passed through 3 × 3 convolution, eliminating the aliasing effect caused by up-sampling. The four prediction feature layers $P_{2}$, $P_{3}$, $P_{4}$ and $P_{5}$ are obtained. The $P_{5}$ layer features are subsampled to obtain the $P_{6}$ layer features, which are only used for the regional proposal network in the Faster R-CNN algorithm. “0.5×” represents the scaling factor applied during spatial downsampling as the backbone network extracts features layer by layer.

**Figure 11:**
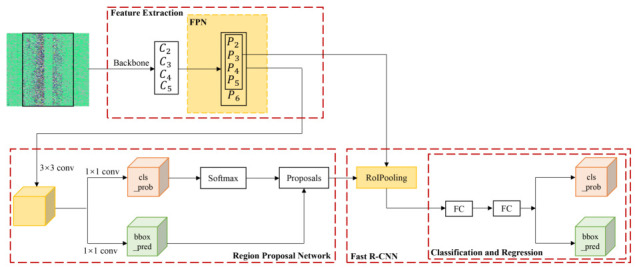
The improved Faster R-CNN based on the FPN.

Figure 11 is the whole network structure of the improved Faster R-CNN based on the FPN. The new model first converts optical fiber signals into intensity images containing spatio-temporal features as input. It utilizes FPN to construct a top-down architecture with lateral connections, fusing deep semantic information with shallow resolution information to generate multi-scale feature maps. Subsequently, it improves the Region Proposal Network so that anchor boxes with corresponding aspect ratios are generated on feature maps at different levels. During the RoI Pooling stage, an adaptive allocation strategy is introduced, which automatically maps candidate regions of different sizes to the most suitable feature level for feature extraction based on a formula. This enables high-precision detection and identification of various complex railway intrusion events. The core architectural innovation lies in the Physics-informed Feature Mapping within the FPN hierarchy. In the context of DAS, the ‘scale’ of an object is determined by its frequency components and spatial impact range. We specifically utilize the high-resolution lateral connections ($P_{2},P_{3}$) to preserve the fine-grained spatial textures of high-frequency micro-vibrations (e.g., thorn-cage pulling or chisel-wall), which are typically discarded in standard CNN down-sampling. Simultaneously, the top-down pathway ($P_{4},P_{5}$) is responsible for capturing the macro-energy evolution of low-frequency, large-scale events. This specialized mapping ensures that the unique spatio-temporal dynamics of DAS signals are fully exploited across different semantic levels. As shown in the yellow part in Figure 11, we introduce the FPN network to optimize the Faster R-CNN algorithm mainly in two parts: the first part is to improve the region proposal network (RPN) at the bottom left of Figure 11. The traditional Faster R-CNN algorithm takes the single-scale feature map output from the feature extraction network as the input of the RPN network and outputs nine rectangular candidate regions with different scales and aspect ratios. In this paper, we use multi-scale feature maps instead of the single-scale feature map, and design each feature map corresponding to one size of anchor in the RPN network to optimize the Faster R-CNN algorithm. To adapt to the railway-specific detection scenario, we optimized the Anchor Box aspect ratios within the RPN. Unlike natural objects, DAS intrusion signals exhibit distinct elongated patterns and specific tilt angles in the spatio-temporal maps, governed by the vibration propagation speed in the subgrade. By constraining the anchors with these physically-derived geometric priors, the model effectively filters out non-target interference and enhances the localization accuracy for linear vibration events. As shown in the bottom diagram of Figure 10, the pixel regions contained by anchor on {$P_{2}$, $P_{3}$, $P_{4}$, $P_{5}$, $P_{6}$} are {$32^{2}$, $64^{2}$, $128^{2}$, $256^{2}$, $512^{2}$} respectively, and three proportion relations (1:1, 1:2, 2:1) are still used. Finally, there are 15 kinds of anchors for each feature map output by FPN. The backbone of the proposed intrusion detection model is constructed using a Faster R-CNN framework enhanced by a Feature Pyramid Network (FPN). Initially, the 1D fiber-optic vibration signals are transformed into 2D spatio-temporal intensity images through a sliding window and linear interpolation process, resulting in a consistent input resolution of 5600×6000 pixels. To effectively capture the multi-scale characteristics of diverse intrusion signatures, the FPN extracts features via a bottom-up pathway, applying a 0.5× spatial downsampling factor at each successive stage. This is followed by a top-down pathway integrated with lateral connections to generate the feature pyramid levels $P_{2}$ through $P_{5}$. In this architecture, shallow but high-resolution features are fused with deep semantic information, enabling the network to simultaneously identify fine-grained high-frequency micro-vibrations, such as thorn-cage pulling, and macro-energy evolutions typical of low-frequency events like mechanical excavation. The second part is to improve the Fast R-CNN algorithm in the bottom right of Figure 11. In the RoI Pooling layer of Fast R-CNN, we set the FPN network to assign different pyramid levels to different scale RoIs, and use different pyramid feature layers as the input of different scale RoIs. The deep feature layer is used for large-scale RoI, and the shallow feature layer is used for small-scale RoI. The final results are used for classification and regression, respectively.

The feature pyramid level *k* assigned to the RoI with width *w* and height *h* can be expressed as [38]:

(7) $$k=\left\lfloor k_{0}+\log_{2}\left(\sqrt{wh}/224\right)\right\rfloor$$

where 224 is the size of the standard pre-training image. $k_{0}$ represents the level of the feature pyramid assigned by the ROI of $w\times h=224^{2}$.

In the Faster R-CNN detection algorithm, $k_{0}$ is set to 4. Intuitively, Equation (7) means that if the RoI’s scale becomes smaller (say, 1/2 of 224), it should be mapped into a finer-resolution level (say, k=3).

To train the improved Faster R-CNN model, we employ a multi-task loss function to jointly optimize classification and bounding box regression. The total loss function *L* is defined as the weighted sum of the RPN loss and the ROI-head loss [39]:

(8) $$L\left(\left\{p_{i}\right\},\left\{t_{i}\right\},\left\{c_{j}\right\},\left\{d_{j}\right\}\right)=L_{RPN}\left(\left\{p_{i}\right\},\left\{t_{i}\right\}\right)+L_{ROI}\left(\left\{c_{j}\right\},\left\{d_{j}\right\}\right)$$

Specifically, the loss for each component is expanded as follows:

(9) $$\begin{array}{cc} L\;= & \frac{1}{N_{cls1}}\sum_{i}L_{cls}\left(p_{i},p_{i}^{*}\right)+\lambda_{1}\frac{1}{N_{reg1}}\sum_{i}p_{i}^{*}L_{reg}\left(t_{i},t_{i}^{*}\right)\\ \; & +\frac{1}{N_{cls2}}\sum_{j}L_{cls}\left(c_{j},c_{j}^{*}\right)+\lambda_{2}\frac{1}{N_{reg2}}\sum_{j}\left[c_{j}^{*}\geq 1\right]L_{reg}\left(d_{j},d_{j}^{*}\right) \end{array}$$

**Design Justification:**

- **Spatio-temporal Correlation:** Since DAS intrusion signals are represented as 2D spatio-temporal images, the $L_{cls}$ (Cross-Entropy) ensures accurate identification of vibration patterns, while $L_{reg}$ (Smooth L1) provides robust localization of the intrusion in both time and space.
- **Multi-scale Adaptation:** In conjunction with the FPN, this joint loss enables the network to maintain high sensitivity to micro-vibrations through shallow-layer features while capturing macro-energy patterns (e.g., train noise) via deep-layer features.
- **Robustness to Background Noise:** The SmoothL1 loss is specifically chosen for $L_{reg}$ because it is less sensitive to outliers compared to L2 loss, which is crucial for handling the non-stationary background noise generated by passing trains.

The idea of the improved Faster R-CNN based on the FPN mainly boosts the performance of the Faster R-CNN feature extraction module. FPN improves the RPN and the Fast R-CNN in the Faster R-CNN algorithm, which enables the Faster R-CNN algorithm to extract the features of multi-scale spatio-temporal signal intensity images, so as to improve the detection accuracy of the Faster R-CNN detection algorithm.

## 4. Experiments and Analysis

**Figure 12:**
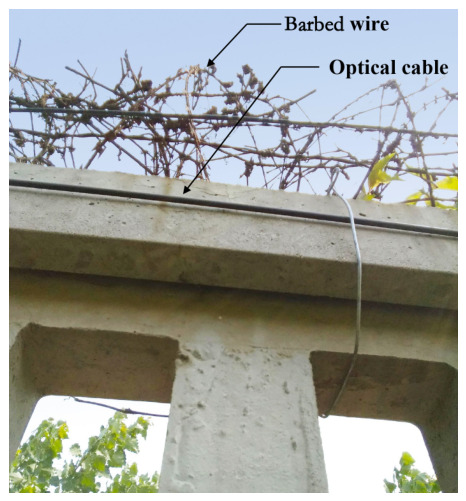
Real experimental scene.

In the experiments, we established the high-speed railway intrusion detection system, where the DAS system based on ϕ-OTDR is deployed around the high-speed railway. With this system, a field trial was conducted on a fence along the high-speed railway to evaluate the performance of the system. As shown in Figure 12, in order to prevent man-made damage to the optical cable, we fix the vibration detection optical cable inside the high-speed railway fence and place barbed wire on it. The DAS-based intrusion detection system has a spatial resolution of 6.8 m, a maximum sensing range of 40 km, and a laser light source with a central wavelength of 1550 nm. The theoretical maximum range of the DAS interrogator is 40 km, and the physical spatial resolution of the system is 6.8 m. The vibration detection optical cable uses the standard G.652 single-mode optical fiber (Yangtze Optical Fibre and Cable Joint Stock Limited Company, Wuhan, China).The signal sampling rate in the optical signal demodulation host is 488 Hz. The experiments are conducted on a server with an Nvidia GeForce GTX 1060 GPU (NVIDIA Corporation, Santa Clara, CA, USA) and the model is trained until the losses were converged.

### Field Data Acquisition

We select multiple sampling points to collect the optical fiber acoustic signals around the high-speed railway. The initial position is based on the chassis, and the acquisition positions of signals are 6.8 m, 108.8 m, 850 m, 4220 m and 4352 m respectively. The considered five types of intrusion events are as follows.

- **Train Background Noise Interference:** Train background noise interference refers to the optical fiber vibration signal when a high-speed train passes. We mark this signal as the non-threatening intrusion interference signal.
- **Pulling Thorn Cage:** Pulling the thorn cage refers to the intruder pulling, cutting, and destroying the thorn cage placed on the high-speed railway fence.
- **Climbing Fence:** Climbing fence refers to the intruder trying to climb over the fence to enter the high-speed railway perimeter, which is the most common intrusion event.
- **Excavating and Chiseling Wall:** Excavating and chiseling wall refers to the intruder using hard objects to stress damage to the high-speed railway fence, resulting in a gap of a certain width at the fence to enter the high-speed railway perimeter.

A total of 779 spatio-temporal signal feature image samples were collected, of which 623 samples were randomly selected as training data, and the rest were used for testing. Figure 8 shows the spatio-temporal intensity images of the six kinds of fiber signals in a certain period. From Figure 8, we can see that the image of each type of fiber optic signal has its unique features that facilitate further identification of intrusion events.

## 5. Evaluation of Detection Model

In the training phase of the model, the default batch size is 1, and 150,000 times of training have been performed. One epoch means that all the samples of the training set participate in the training of model once. The initial learning rate is set to 0.001. The loss function is the sum of the classification loss and the regression loss.

Table 2 shows the final prediction results of the test set. Table 3 and Table 4 provides a comprehensive performance comparison between our proposed FPN-based Faster R-CNN and several state-of-the-art method. The performance is evaluated across five specific intrusion categories and background noise using three standard metrics: Precision (P), Recall (R), and F1-score (F1). These metrics offer a balanced assessment of the system’s ability to correctly identify threats while minimizing false alarms. As shown in Table 2, we analyzed the experimental results in detail, including precision, recall, and $F_{1}$ score.

- Precision: The proportion of samples with correct prediction. It can be calculated by
  (10) $$Precision=\frac{TP}{TP+FP}$$
- Recall: The proportion of samples that are actually predicted to be positive objects among all positive objects. It can be expressed as
  (11) $$Recall=\frac{TP}{TP+FN}$$
- $F_{1}$ score: The metric represents the equilibrium relationship of precision and recall. We have
  (12) $$F_{1}=\frac{2\times Precision\times Recall}{Precision+Recall}$$

In the above index descriptions, True Positive (TP), False Positive (FP), and False Negative (FN) denote positive samples detected correctly, negative samples detected incorrectly, and positive samples detected incorrectly, respectively.

As shown in Table 2, we tested 156 samples. The model detects five kinds of intrusion events with a precision of over 91%. Due to the spatio-temporal signal feature intensity images of the climbing fence and pulling thorn cage being similar, a few samples are misclassified. Overall, the average detection accuracy of the test set can reach 95.51%.

**Table 2:** Test set samples prediction result.

Intrusion Events		Prediction Label					Precision	Recall	$F_{1}$-Score
		1	2	3	4	5			
True Label	Climbing	31	0	2	0	0	0.939	0.939	0.939
	Chiselwall	1	39	0	1	0	0.975	0.951	0.963
	Pullthorncage	1	0	22	0	0	0.917	0.957	0.936
	Excavate	0	0	0	38	1	0.974	0.974	0.974
	Traininterference	0	1	0	0	19	0.950	0.950	0.950
Detection average accuracy		95.51%					—		

Furthermore, in order to better and rationally quantify the performance of the detection model, we compared the method proposed in this paper with the methods described in [19,22,24,25,26,30]. The three baseline systems are as follows:

- **ConvLSTM:** The method is based on the combination of CNN and long short-term memory (LSTM) networks to detect intrusion events in the railway detection system.
- **Reinforce-ConvLSTM:** Based on ConvLSTM method, the method can distinguish noise and interference effectively for high-speed railway intrusion detection.
- **RT-DETR:** The RT-DETR model, through its efficient hybrid encoder and end-to-end detection architecture, maintains high real-time inference performance while effectively enhancing multi-scale feature fusion and recognition for railway intrusion targets. The method can distinguish noise and interference effectively for high-speed railway intrusion detection.
- **MSIA-YOLOv8:** By integrating a multi-scale interactive attention mechanism and a frequency domain aggregation and enhancement (FDAE) module, it effectively extracts features of distant small objects under complex railway backgrounds, significantly improving recognition accuracy and robustness. The method can distinguish noise and interference effectively for high-speed railway intrusion detection.
- **Rail-STrans:** Based on an improved Swin Transformer, it utilizes a hierarchical architecture and a shifted window mechanism to capture global context information, enabling precise localization and fine-grained segmentation of minute rail surface defects. The method can distinguish noise and interference effectively for high-speed railway intrusion detection.
- **Faster R-CNN:** The method uses the DAS system with the Faster R-CNN algorithm to detect and identify railway intrusion events.

The comparison results are shown in Table 3, Table 4 and Table 5. From this Table, we can obtain the following conclusions.

- Our proposed method achieves the best detection performance. Compared with the other methods, the proposed method can detect and identify more types of intrusion events and the $F_{1}$ score of each intrusion event is above 93%. Compared with the study in [19], the model achieves higher detection accuracy due to the newly added FPN structure, which improves the problem of insufficient feature information extracted by the detection algorithm.
- In terms of inference speed (Table 5), our method achieves 27.5 FPS (36.4 ms) on an NVIDIA GTX 1060. While this is slightly lower than the one-stage RT-DETR (80.6 FPS), our method provides better stability in complex railway environments. The “slight latency” compared to one-stage models is a deliberate trade-off: the two-stage refinement process ensures higher precision in signal localization, which is more critical for railway safety than extreme high-speed processing. Our proposed method can identify the background noise interference generated by passing trains, and the detection accuracy is 95%. Compared with the study in [19,22], the model proposed in this paper has better performance in identifying the noise interference event, which can significantly reduce the false alarm rate of the system.

**Table 3:** Performance comparison of proposed method with previous methods (Part I). “$F_{1}$” stands for $F_{1}$ score.

Method	Intrusion Event Detection								
	Climb			Chiselwall			Pullthorncage		
	P	R	*F*_1_	P	R	*F*_1_	P	R	*F*_1_
ConvLSTM [22]	85.44%	86.04%	85.74%	84.09%	94.31%	88.91%	84.29%	75.64%	79.73%
Reinforce-ConvLSTM [30]	87.40%	87.10%	87.25%	91.91%	93.85%	92.86%	82.35%	84.56%	83.44%
RT-DETR [24]	93.82%	92.47%	93.16%	96.25%	94.55%	94.52%	93.21%	91.83%	92.50%
MSIA-YOLOv8 [25]	91.50%	90.20%	90.8%	93.80%	91.51%	92.64%	90.85%	89.63%	90.22%
Rail-STrans [26]	90.22%	89.17%	89.66%	92.13%	90.88%	91.47%	89.59%	88.43%	88.90%
Faster R-CNN [19]	91.34%	91.18%	91.26%	91.67%	90.79%	91.23%	90.50%	92.22%	91.35%
Proposed method	93.94%	93.94%	93.94%	97.50%	95.12%	96.30%	91.67%	95.65%	93.62%

**Table 4:** Performance comparison of proposed method with previous methods (Part II). “-” means unreported.

Method	Intrusion Event Detection					
	Excavate			Traininterference		
	P	R	*F*_1_	P	R	*F*_1_
ConvLSTM [22]	-	-	-	-	-	-
Reinforce-ConvLSTM [30]	-	-	-	-	-	-
RT-DETR [24]	96.53%	95.02%	95.71%	95.22%	93.52%	94.33%
MSIA-YOLOv8 [25]	94.21%	92.86%	93.50%	93.51%	91.88%	92.64%
Rail-STrans [26]	92.53%	91.27%	91.82%	92.81%	90.53%	91.63%
Faster R-CNN [19]	91.77%	91.19%	91.48%	90.41%	92.08%	91.24%
Proposed method	97.44%	97.44%	97.44%	95.00%	95.00%	95.00%

**Table 5:** Inference Time of proposed method with previous methods.

Method	Device	Inference Time (ms)	FPS
ConvLSTM [22]	NVIDIA GTX 1060	68.4	14.6
Reinforce-ConvLSTM [30]	NVIDIA GTX 1060	62.1	16.1
RT-DETR [24]	NVIDIA GTX 1060	28.5	35.1
MSIA-YOLOv8 [25]	NVIDIA GTX 1060	32.7	30.6
Rail-STrans [26]	NVIDIA GTX 1060	45.9	21.8
Faster R-CNN [19]	NVIDIA GTX 1060	145.2	6.9
Proposed Method	NVIDIA GTX 1060	36.4	27.5

**Figure 13:**
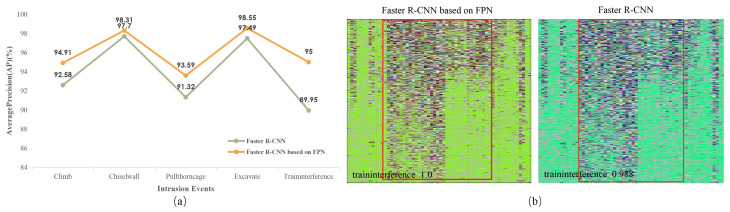
(**a**,**b**) Detect average precision of high-speed railway intrusion events for two methods.

We also compared the average precision of the proposed method in this paper with the method described in [19]. As shown in Figure 13, by applying the FPN to the Faster R-CNN algorithm, the average detection precision of the model for the five kinds of railway intrusion events can be further improved.

In practical high-speed railway environments, seasonal and diurnal temperature variations can affect the fiber optic sensing system. Specifically, temperature fluctuations lead to changes in the fiber’s refractive index and physical length, which may cause slow drifts in the phase-sensitive optical time-domain reflectometry (ϕ-OTDR) signal.

However, our system mitigates these effects through two primary mechanisms:

- First, the DAS system employs a high-speed differential signal processing method that subtracts adjacent traces, effectively canceling out slow-varying environmental drifts like temperature.
- Second, the deep learning model (FPN-based Faster R-CNN) focuses on the high-frequency spatio-temporal vibration patterns of intrusion events, which are intrinsically distinct from the quasi-static signal changes caused by temperature variations.

Therefore, the system maintains stable detection performance across different environmental conditions.

## 6. Conclusions

In this paper, an FPN-based Faster R-CNN framework is proposed to address the challenge of high-precision intrusion detection along high-speed railways using DAS. The primary advantage of the proposed FPN-based Faster R-CNN system lies in its ability to bridge the gap between 1D physical acoustic signals and 2D deep learning arc (FPN), the system effectively aligns multi-scale feature fusion with the physical propagation characteristics of railway intrusions. Unlike traditional single-scale detection methods, our approach demonstrates superior robustness against the intense, non-stationary background noise generated by passing trains, achieving a high average accuracy of 95.51% with a real-time inference latency of only 36.4 ms. However, certain limitations remain: (1) The diversity of the dataset could be further enriched by incorporating signals under extreme weather conditions to improve robustness. (2) While the detection accuracy is high, the model’s deployment on resource-constrained edge computing devices for real-time processing remains a subject for future work.

## Figures and Tables

**Table 1:** Comparison of different intrusion detection technologies for high-speed railway security.

Technology	Monitoring Range	Positioning Accuracy	Cost-Efficiency
Video Surveillance (CCTV) [3]	Short (∼100 m)	N/A	Low (High maintenance)
Microwave Radar [4]	Medium (∼500 m)	±1–5 m	Very Low (High device cost)
Vibration Cable [5]	Medium (∼1 km)	±10–50 m	Medium
Proposed DAS	Long (Up to 40 km)	±5–10 m	High (Fiber reuse)

## Data Availability

The data presented in this study are available on request from the corresponding author. The data are not publicly available due to privacy restrictions. In this paper, a high-precision fiber DAS intrusion detection system is proposed. We collect the optical fiber acoustic signals using the DAS system based on ϕ-OTDR and propose a novel framework that integrates the FPN and the Faster R-CNN to adaptively extract the spatio-temporal features from the fiber signal intensity images. The experimental results prove that the system can accurately detect and identify five types of high-speed railway perimeter intrusion events, and its performance is better than the previous methods. Additionally, the system can accurately identify the background noise interference generated by passing trains. For future work, we plan to improve the performance of the proposed system by multimodal fusion of distributed fiber optic sensors with video sensors.

## Abbreviations

The following abbreviations are used in this manuscript:

DAS	Distributed Acoustic Sensing
FPN	Feature Pyramid Network
R-CNN	Region-based Convolutional Neural Network
RoI	Region of Interest
RPN	Region Proposal Network
ResNet	Residual Network
SNR	Signal-to-Noise Ratio
ReLU	Rectified Linear Unit
IoU	Intersection over Union
AP	Average Precision
mAP	mean Average Precision
P	Precision
R	Recall
F1-score	F-measure
EDFA	Erbium-Doped Fiber Amplifier
AOM	Acousto-Optic Modulator
BPF	Band-Pass Filter
FPGA	Field Programmable Gate Array

## References

[1] Chen R., Long W.X., Mao G., Li C. (2018). Development Trends of Mobile Communication Systems for Railways. IEEE Commun. Surv. Tutor..

[2] Bernal E., Spiryagin M., Cole C. (2019). Onboard Condition Monitoring Sensors, Systems and Techniques for Freight Railway Vehicles: A Review. IEEE Sens. J..

[3] Zou Z., Chen K., Shi Z., Guo Y., Ye J. (2023). Object Detection in 20 Years: A Survey. Proc. IEEE.

[4] Yanwei J., Yu D. Research on Railway Obstacle Detection Method Based on Radar. Proceedings of the 2021 7th International Symposium on Mechatronics and Industrial Informatics (ISMII).

[5] Xu H., Li Y., Ma C., Liu L., Wang B., Li J. (2022). A combined sensing system for intrusion detection using anti-jamming random code signals. Sensors.

[6] Hodge V.J., O’Keefe S.E.M., Weeks M., Moulds A. (2015). Wireless Sensor Networks for Condition Monitoring in the Railway Industry: A Survey. IEEE Trans. Intell. Transp. Syst..

[7] Juarez J., Taylor H. (2005). Distributed fiber-optic intrusion sensor system. J. Light. Technol..

[8] Martins H.F., Martín-López S., Corredera P., Filograno M.L., Frazão O., González-Herráez M. (2014). Phase-sensitive Optical Time Domain Reflectometer Assisted by First-order Raman Amplification for Distributed Vibration Sensing over >100 km. J. Light. Technol..

[9] Tejedor J., Martins H., Piote D., Macias-Guarasa J., Pastor-Graells J., Martín-López S., Guillen P.C., Smet F.D., Postvoll W., González-Herráez M. (2016). Toward Prevention of Pipeline Integrity Threats Using a Smart Fiber-Optic Surveillance System. J. Light. Technol..

[10] Wang Z.N., Li J., Fan M., Zhang L., Peng F., Wu H., Zeng J., Zhou Y.X., Rao Y.J. (2014). Phase-sensitive optical time-domain reflectometry with Brillouin amplification. Opt. Lett..

[11] Shi Y., Feng H., Zeng Z. (2015). A Long Distance Phase-Sensitive Optical Time Domain Reflectometer with Simple Structure and High Locating Accuracy. Sensors.

[12] Liang S., Sheng X., Lou S., Feng Y., Zhang K. (2016). Combination of Phase-Sensitive OTDR and Michelson Interferometer for Nuisance Alarm Rate Reducing and Event Identification. IEEE Photonics J..

[13] Ma P., Liu K., Jiang J., Li Z., Li P., Liu T. (2018). Probabilistic Event Discrimination Algorithm for Fiber Optic Perimeter Security Systems. J. Light. Technol..

[14] Jia H., Liang S., Lou S., Sheng X. (2019). A *k*-Nearest Neighbor Algorithm-Based Near Category Support Vector Machine Method for Event Identification of *ϕ*-OTDR. IEEE Sens. J..

[15] Zhong X., Zhao S., Deng H., Gui D., Zhang J., Ma M. (2020). Nuisance alarm rate reduction using pulse-width multiplexing *Φ*-OTDR with optimized positioning accuracy. Opt. Commun..

[16] Wu H., Liu X., Xiao Y., Rao Y. (2019). A Dynamic Time Sequence Recognition and Knowledge Mining Method Based on the Hidden Markov Models (HMMs) for Pipeline Safety Monitoring with *ϕ*-OTDR. J. Light. Technol..

[17] Sun Q., Feng H., Yan X., Zeng Z. (2015). Recognition of a Phase-Sensitivity OTDR Sensing System Based on Morphologic Feature Extraction. Sensors.

[18] Wu H., Xiao S., Li X., Wang Z., Xu J., Rao Y. (2015). Separation and Determination of the Disturbing Signals in Phase-Sensitive Optical Time Domain Reflectometry (*Φ*-OTDR). J. Light. Technol..

[19] Xiao X., Ma X., Hui Y., Yin Z., Luan T.H., Wu Y. Intrusion Detection for High-speed Railway System: A Faster R-CNN Approach. Proceedings of the 2021 IEEE 94th Vehicular Technology Conference (VTC2021-Fall).

[20] Wu H., Chen J., Liu X., Xiao Y., Wang M., Zheng Y., Rao Y. (2019). One-Dimensional CNN-Based Intelligent Recognition of Vibrations in Pipeline Monitoring with DAS. J. Light. Technol..

[21] Aktas M., Akgun T., Demircin M.U., Buyukaydin D. Deep learning based multi-threat classification for phase-OTDR fiber optic distributed acoustic sensing applications. Proceedings of the Commercial + Scientific Sensing and Imaging.

[22] Li Z., Zhang J., Wang M., Zhong Y., Peng F. (2020). Fiber distributed acoustic sensing using convolutional long short-term memory network: A field test on high-speed railway intrusion detection. Opt. Express.

[23] Shiloh L., Eyal A., Giryes R. (2018). Deep Learning Approach for Processing Fiber-Optic DAS Seismic Data. Optical Fiber Sensors 2018.

[24] Li P., Peng Y., Wang S.M., Zhong C. (2025). Improved RT-DETR Framework for Railway Obstacle Detection. IEEE Access.

[25] Hu T., Gao F., Zhou F. (2026). Railway obstacle intrusion detection and risk assessment based on MSIA-YOLOv8 and DALNet. Expert Syst. Appl..

[26] Si C., Luo H., Han Y., Ma Z. (2024). Rail-STrans: A Rail Surface Defect Segmentation Method Based on Improved Swin Transformer. Appl. Sci..

[27] Taylor H.F., Lee C.E. (1993). Apparatus and Method for Fiber Optic Intrusion Sensing. U.S. Patent.

[28] Sifta R., Munster P., Sysel P., Horváth T., Novotný V., Krajsa O., Filka M. (2015). Distributed Fiber-Optic Sensor for Detection and Localization of Acoustic Vibrations. Metrol. Meas. Syst..

[29] Li Q., Zhang C., Li L., Zhong X. (2014). Localization mechanisms and location methods of the disturbance sensor based on phase-sensitive OTDR. Optik.

[30] Li Z., Zhang J., Wang M., Chai J., Wu Y.X., Peng F. (2020). An anti-noise *ϕ*-OTDR based distributed acoustic sensing system for high-speed railway intrusion detection. Laser Phys..

[31] Ren S., He K., Girshick R.B., Sun J. (2015). Faster R-CNN: Towards Real-Time Object Detection with Region Proposal Networks. IEEE Trans. Pattern Anal. Mach. Intell..

[32] Dai J., Li Y., He K., Sun J. (2016). R-FCN: Object Detection via Region-based Fully Convolutional Networks. arXiv.

[33] Zhang J., Hu H., Chen S., Huang Y., Guan Q. Cancer Cells Detection in Phase-Contrast Microscopy Images Based on Faster R-CNN. Proceedings of the 2016 9th International Symposium on Computational Intelligence and Design (ISCID).

[34] Fan Q., Brown L., Smith J. A closer look at Faster R-CNN for vehicle detection. Proceedings of the 2016 IEEE Intelligent Vehicles Symposium (IV).

[35] Le T., Zheng Y., Zhu C., Luu K., Savvides M. Multiple Scale Faster-RCNN Approach to Driver’s Cell-Phone Usage and Hands on Steering Wheel Detection. Proceedings of the 2016 IEEE Conference on Computer Vision and Pattern Recognition Workshops (CVPRW).

[36] Wu W., Yin Y., Wang X., Xu D. (2019). Face Detection with Different Scales Based on Faster R-CNN. IEEE Trans. Cybern..

[37] Shao C., Zhang L., Pan W. (2021). Faster R-CNN Learning-Based Semantic Filter for Geometry Estimation and Its Application in vSLAM Systems. IEEE Trans. Intell. Transp. Syst..

[38] Lin T.Y., Dollár P., Girshick R.B., He K., Hariharan B., Belongie S.J. Feature Pyramid Networks for Object Detection. Proceedings of the 2017 IEEE Conference on Computer Vision and Pattern Recognition (CVPR).

[39] Jiang H., Learned-Miller E. Face Detection with the Faster R-CNN. Proceedings of the 2017 12th IEEE International Conference on Automatic Face & Gesture Recognition (FG 2017).

